# University Student’s Academic Goals When Working in Teams: Questionnaire on Academic Goals in Teamwork, 3 × 2 Model

**DOI:** 10.3389/fpsyg.2019.02434

**Published:** 2019-10-24

**Authors:** Benito León-del-Barco, Santiago Mendo-Lázaro, Ma Isabel Polo-del-Río, Irina Rasskin-Gutman

**Affiliations:** Department of Psychology and Anthropology, University of Extremadura, Cáceres, Spain

**Keywords:** academic goals, motivation, teamwork, university, students

## Abstract

Group work is a very common practice in higher education when it comes to developing key competences for students’ personal and professional growth. The goals that students pursue when working in teams determine how they organize and regulate their behavior and how they approach the tasks. The academic goals are a relevant variable that can condition the success of the group, as they guide and direct the students toward involvement in the task, the effort they make, and the desire to increase their academic competence, and their learning. Thus, the need arises to create new evaluation instruments to help us understand the importance of academic goals when students work as a team. The purpose of this paper is to corroborate the construct validity of the *questionnaire on teamwork learning goals* (QTLG) based on the *achievement goal questionnaire* (3 × 2 AGQ) of Elliot et al. (2011) in the context of teamwork, and to determine if the model 3 × 2 offers a better fit to the data than other models, such as: 2 × 2; Trichotomous; Definition; Valence, among others. The results obtained from a sample of 700 students from 6 Spanish universities confirm that, in the context of teamwork, the 3 × 2 model fits the data better than the rest of the models subjected to confirmatory analysis, with contrasting evidence of validity and reliability. Therefore, we considered it a useful instrument for studying motivation in the group work context. The QTLG has practical applications, allowing us to explore in detail the academic goals of university students.

## Introduction

Adaptation to the European higher education area (EHEA) has brought about an important methodological revolution: the allocation of new meanings to learning and teaching activities (González and Raposo, 2008). In accordance with this new approach, rather than teaching processes and the instructor’s work in the classroom, today it is the learning process through which students achieve their proposed goals in each subject that are of interest (Palacios, 2004; Prichard et al., 2006). In this sense, the implementation of active methodologies that encourage and reinforce both autonomous learning and teamwork, has facilitated the development of fundamental competences, such as the organization of information, communication, conflict management. All of these competences are essential for students’ personal and professional growth (Manzer and Bialik, 1997; Blignaut and Venter, 1998; Gottschall and García-Bayonas, 2008; Gaudet et al., 2010; Mendo et al., 2016; León et al., 2017).

While group work is a very common practice in higher education, it is not always a satisfactory experience for the students (Payne and Monk-Turner, 2006; Burdett, 2007). To avoid the problems that underlie group work, instructors should, on the one hand, keep up to date with methodologies that favor cooperation among peers and, on the other, advise their students about how to acquire the necessary social skills for teamwork, for instance: active listening, assertiveness, expressing opinions, arguments, criticism, and praise (Rodríguez and Ridao, 2014; León et al., 2015). Other elements to be taken into account are the composition of the team, its cohesion, the group-class atmosphere, conflict management, the group’s beliefs concerning the perception and effectiveness of their performance, how the task has been designed and interdependence (León et al., 2017, 2018), evaluation (Beigi and Shirmohammadi, 2012), and attitudes toward teamwork (Mendo et al., 2017). Similarly, we also believe that the goals students pursue when working in teams is a relevant variable that may condition the group’s success.

In the educational sphere, learning goals refer to what guides students toward becoming involved in the task, making an effort, and increasing a desire for academic competence and learning. For Locke and Latham (1990), goals provide guidance and focus attention on the task being carried out and activate and enhance the effort, increasing the time dedicated and promoting the development of new learning strategies when the proposed goals are not achieved.

### Academic Goal Types

Two types of goals have traditionally been considered in the study of academic goals, learning goals and performance goals. On the one hand, when students wish to acquire knowledge, increase their competence, master and enjoy the task, in short, learn and improve their skills, these are learning goals, also known as domination and task-centered goals (Nicholls and Miller, 1984; Dweck, 1986; Shim et al., 2013). On the other hand, when students wish to perform well academically, advance in their studies, appear highly competent before others and/or avoid being seen as incompetent, i.e., they seek positive assessments concerning their performance, we are referring to performance goals, also described as execution goals or self-centered goals (Nicholls and Miller, 1984; Dweck, 1986; Elliot, 1999).

From this two-dimensional approach to a three-dimensional approach (Elliot and Harackiewicz, 1996) and a 2 × 2 model (Elliot and McGregor, 2001; Elliot, 2005), we have come to a 3 × 2 model (Elliot et al., 2011). Atkinson (1964) pointed out that everyone feels the need to be successful and avoid failure. The resulting motivation usually pushes us to take risks in order to achieve the desired success or avoid certain situations. The three-dimensional model (Elliot and Harackiewicz, 1996) considers the difference between approach goals and avoidance goals (direction of the goal) within performance goals. The 2 × 2 model (Elliot and McGregor, 2001; Elliot, 2005) adds the difference between the approach or avoidance goals and learning goals (orientation of the goal). The tendency toward avoidance in learning goals had little theoretical clarity, despite the fact that motivation experts have examined the existence of a tendency toward approach, which leads people toward positive final states, and another tendency toward avoidance, which leads them away from negative final states (Mega et al., 2014). The tendency toward avoidance in learning goals helped to clarify early inconsistencies in performance goals findings (Midgley et al., 2001; Brophy, 2005; Elliot et al., 2011; Murayama et al., 2011). The difference between approach goals or avoidance goals (mastery-approach, performance-approach, mastery-avoidance, and performance-avoidance) is now widely accepted by researchers. Objectives based on avoidance focus on failure, and regulation involves trying to stay away or stay away from this negative possibility (Elliot et al., 2011). Avoidance motivation has been associated with lower academic performance and negative emotions (Huang, 2012).

Finally, the 3 × 2 model (Elliot et al., 2011), in the words of Lower and Turner (2016), aims to improve the accuracy of the 2 × 2 model by considering various references, such as *task*, *self* and *other*, in the evaluation of students’ competences (*My goals in the exams of the subjects I am studying are*…). The definition of competence depends on the model used to determine whether we are doing something right or wrong. In task-based goals, the task itself is used as a reference point; in the case of self-based goals, the competence is defined in terms of doing better or worse than on other occasions – the reference is past personal experience – and, in the case of other-based goals, the competence is defined in terms of doing things better or worse than the others.

In this way, some *task-based goals* are achieved with a tendency toward approach or avoidance (“*To have many correct answers*”*;* “*To avoid incorrect answers*”); goals based on self (“*Do better than I did in the previous exams*”*;* “*Avoid doing worse than in the previous exams*”); and goals based on the other, similarly with the orientation toward approach or avoidance (“*Do better than my classmates*”*;* “*Avoid doing worse than my classmates*”). Elliot et al. (2011) base the model on the data obtained in the *achievement goal questionnaire* (3 × 2 AGQ) from a sample of university students.

### Key Elements to Teamwork

Although there is evidence of the influence of learning goals, less is known about their influence on teamwork groups. Mercier (2017) suggests that achievement goals influence interaction behaviors when students are engaged in group activities. University students pursue their own academic goals; nevertheless, the study of such goals is of interest when they participate in teamwork. The goals pursued by students as part of a team are important because they determine how they organize and regulate their behavior and how they approach group tasks. Teamwork entails organized collaboration in order to achieve a common objective (Johnson et al., 1999). Each student in the group has their particular knowledge, skills and goals that come into play in pursuit of the said objective.

Similarly, a positive attitude toward teamwork, i.e., the belief that success would not be possible without the effort of all the group members, is fundamental in predicting success regarding learning (Hijzen et al., 2006). There is clear evidence of a relationship between the expectations of team performance and attitudes toward teamwork, especially with regard to social and affective attitudes. Both motivational variables – team potency and attitudes toward learning teams – are related to group efficacy (Castelló, 1998; León et al., 2017; Mendo et al., 2017).

Another relevant variable in predicting group effectiveness and performance is the group’s potency, a concept originally defined by Guzzo et al. (1993), which refers to the group’s collective belief that the group can be effective; it is an essential construct related to group motivation.

Several research projects have focused their attention on variables that are fundamental when evaluating the effectiveness of teamwork. Hence, we point to the studies of Pfaff and Huddleston (2003), Gottschall and García-Bayonas (2008), Alford et al. (2014) in relation to the preference or evaluation of teamwork experiences and the studies of Beigi and Shirmohammadi (2012), concerning evaluation and the work environment. These studies have found that concerns about the assessment outcomes of teamwork and the perceptions of the teamwork environment affect students’ attitudes toward teamwork.

Other studies, such as those made by León et al. (2017) or Collins and Parker (2010), show that there is solid evidence of the importance of team potency. In a meta-analysis of 67 studies, positive and moderate relations were shown between team potency and group performance (Gully et al., 2002).

Finally, the work done by Kouros and Abrami (2006) and Nausheen et al. (2013), on the quality of the product and the process, the support of teammates, interdependence and frustration, confirm the direct and positive relationship between the interdependence and effectiveness of teamwork (Mena et al., 2012). The research on the interdependence and effectiveness of teamwork in organizations exposes the modulating effect that the task interdependence has shown on many of the effects that different team processes have on organizational outcomes, i.e., assisting behaviors, confidence, communication, and conflict or flexibility (Rico et al., 2010).

### The Current Research

Echoing the desire of Elliot et al. (2011) to keep the study of academic achievement goals in the vanguard of research into motivation, and applying the 3 × 2 model to other, different evaluative environments (e.g., teamwork) on how students face the exams in a specific subject; we decided to analyze the academic goals when working in teams. The first step to know the goals pursued by students working in teams is to create evaluation instruments. So, in this research, our aim is to evaluate goals when working as a team using the 3 × 2 model of Elliot et al. (2011). To this end, starting from the *achievement goal questionnaire* (3 × 2 AGQ), we have created the *questionnaire on teamwork learning goals.*

We believe that the design of instruments to evaluate and define these variables for the university context in group learning situations will provide instructors with fundamental diagnostic and evaluation information that can improve how workgroups operate as well as their effectiveness.

Méndez-Giménez et al. (2017) have verified the structural validity of the Spanish version of the achievement goal questionnaire (3 × 2 AGQ) of Elliot et al. (2011) using a sample of 2,630 non-university Spanish students studying in secondary schools and high schools.

The purpose of this paper is to corroborate the construct validity of the 3 × 2 model in the context of teamwork. The idea is to determine whether the existence of the six goals of the 3 × 2 model correspond to solid and well-defined factors and offer a better fit to the data than the 2 × 2 and Trichotomous models and other models, such as the definition or valence models. Regarding the definition models, when considering the referents of task, self and other, we would obtain several models consisting of a single factor, either task, self or other and four factors in the valence, approach or avoidance models (e.g., Approach task or Avoidance task with 5 factors. The items that define the task are loaded in a single factor, the rest of the items are loaded in their respective hypothetical factors; F1 Approach-Avoidance-Task, F2 Approach-Self, F3 Avoidance-Self, F4 Approach-Other, and F5 Avoidance-Other).

Regarding valence models, Elliot (1999) considers that the approach-avoidance valence is the basis on which motivation can be distinguished. In the case of approach motivation, behavior is instigated or guided by a desirable and positive possibility (success), while in the case of avoidance motivation, behavior is instigated or guided by a negative and undesirable possibility (failure). Several models would be obtained by considering the items that define the approach or avoidance trend for Task, Self, and Other as a single factor (e.g., 4-factor avoidance model, the items that define the approach trend refer to the three hypothetical factors of Task, Self, and Other and all the items that define the avoidance trend refer to a single common factor; F1 Approach-Task, F2 Approach-Self, F3 Approach-Other, and F4 Avoidance-Task-Self-Other).

Finally, we shall assess the gender invariance of the 3 × 2 model. Additionally, in order to establish its nomological validity in the context of teamwork, we shall analyze the relationships among the six academic goals with attitudes toward teamwork and team potency; relevant variables when predicting team effectiveness related to group motivation.

## Materials and Methods

### Participants

This study involved 700 students (63% female and 37% male) aged between 18 and 55, with an average age of 21.23 (*SD* = 4.98). The selection of students was carried out by means of a cluster sampling technique that consisted in selecting 6 Spanish public universities at random (Extremadura, Huelva, Valladolid, Granada, Salamanca, and the Complutense of Madrid). Regarding their grade, 49% of the students were in their first year, 26% in their second, 20% in their third and 5% in their fourth year. The students were taking degrees in Infant Education, Primary Education, Social Education, Sociology, Social Work, and Psychology. Students in these degree courses were chosen because of the large amount of course content and evaluable activities related with teamwork that they had to do from the very beginning of their university degrees; thus, ensuring that they had been involved in teamwork in a university environment.

### Instruments

#### Questionnaire on Teamwork Learning Goals (QTLG)

The questionnaire on teamwork learning goals evaluates the academic goals pursued by the students when working in groups. It is an adaptation of the *achievement goal questionnaire* (3 × 2 AGQ) of Elliot et al. (2011). We have used the version translated from the AGQ by Méndez-Giménez et al. (2017), to which we have added a new heading: “*When I carry out a task or work in a group, my goals are.*” We have adapted the contents of the items to this new heading so that the statements of each item evaluate the types of academic goals that the students pursue when working as a team (see Supplementary Material). The QTLG is made up of 18 items that are rated on a 7-point Likert-type scale from 1 (not at all true for me) to 7 (very true for me).

As in the *Achievement Goal Questionnaire*, the QTLG determines six academic goals derived from the combination of three referents, which are the task, the self and the other, as well as the tendency toward approach or avoidance. We thus obtain some goals based on the task with the approach or avoidance tendency (“*Do well in many tasks or work in all subjects*”, “*Avoid doing poorly in tasks or work in all subjects*”); some goals based on self (“*Do better than I did in the past in teamwork*”, “*Avoid doing worse than I usually do in teamwork*”); and some goals based on other, similarly with the approach or avoidance orientation (“*Do better than the other students*”, “*Avoid doing worse than the other students*”).

#### The Questionnaire on Attitudes Toward Learning Teams (QALT) (Mendo et al., 2017)

The questionnaire on attitudes toward learning teams evaluates attitudes toward teamwork. It is made up of 12 Likert-type items with five numerical intervals ranging from 1 (Totally disagree) to 5 (Totally agree). The QALT is composed of two factors: The first, “*Academic attitudes*” (6 items), explains 32% of the variance and covers the assessment of the academic consequences arising from teamwork. The second factor, “*Social & affective attitudes*” (6 items), explains 30% of the variance and covers the assessment of interactions with colleagues during teamwork. These two factors present a correlation of 0.720 (*p* < 0.001). The alpha (α = 0.91), composite reliability (CR = 0.93), and McDonald’s Omega (Ω = 0.92) indices show that the QALT has good overall reliability and average variance extracted (AVE = 0.65). Both factors of the questionnaire present an adequate level of reliability and an AVE > 0.50: F1 (α = 0.88, CR = 0.88, Ω = 0.85, AVE = 0.59); F2 (α = 0.83, CR = 0.80, Ω = 0.82, AVE = 0.51).

#### Learning Team Potency Questionnaire (LTPQ) (León et al., 2017)

The learning team potency questionnaire evaluates the perception that students have about whether their teamwork group can function successfully regarding the activities carried out in the various subjects. It is made up of 8 Likert-type items with ten numerical intervals ranging from 1 (Totally disagree) to 10 (Totally agree). The LTPQ consists of two factors: the first, C*onfidence* (4 items), evaluates the expectations the students have concerning the effectiveness of their own group. The second factor, *Performance* (4 items), evaluates the students’ perception of whether their work group can successfully perform a set of academic tasks. Some items are: F1“*It is easy for my team to carry out any activity required in the different subjects*”, F2 “*The group work carried out by my team is of high quality.*” The alpha (α = 0.91), Composite Reliability (CR = 0.93) and McDonald’s Omega (Ω = 0.92) indices show that the LTPQ presents good overall reliability and average variance extracted (AVE = 0.65). Both factors display adequate reliability and AVE > 0.50: F1 (α = 0.88, CR = 0.88, Ω = 0.85, AVE = 0.59); F2 (α = 0.83, CR = 0.80, Ω = 0.82, AVE = 0.51).

### Procedure

Initially, an online link to the QALT, LTPQ, and QTLG questionnaires was created using Google Docs applications. Participants (*n* = 700) were contacted during the 2016/2017 academic year. The questionnaires were filled in anonymously, guaranteeing the confidentiality of the data obtained and their exclusive use for research. Insofar as ethical rules are concerned, we followed the ethical guidelines of the American Psychological Association (APA, 2009). All participants provided their written informed consent through a checkbox on the online platform. The study was approved by the Ethics Committee of the University of Extremadura.

### Data Analysis

First, we proceeded to detect the outliers by applying the Mahalanobis distance using the *Tests for normality and outliers* option of the AMOS program. The scatter plots of the residuals carried out showed that there is linearity among the estimated variables. Once we confirmed that the assumptions of linearity and normality of all the observed variables included in the models had been accomplish (Jöreskog and Sörbom, 1996), we performed the confirmatory factor analyses (CFA) using the maximum likelihood method.

To determine whether the model fit the data properly, we used the following goodness-to-fit indices: chi-square probability (χ^2^), χ^2^ divided by degrees of freedom (CMIN/*gl*), *comparative fit index* (CFI), *Tucker-Lewis index* (TLI), *root mean square error of approach* (RMSEA), *and the* standardized root mean square residual (SRMR). As parsimony adjustment measures, we used the *AIC (Akaike Information Criterion)*, and the *BBC (Browne-Cudeck Adjustment Criterion)*. We also used the *bootstrap* method and performed an invariant analysis by gender. Finally, the nomological validity was checked using Pearson correlations and linear regression analysis.

## Results

### Confirmatory Factorial Analysis of the QTLG

We performed a confirmatory factorial analysis (CFA) which, as indicated by Henson and Roberts (2006), is a good practice for the psychometric study of a questionnaire, while also allowing the factorial structure to be confirmed. To carry out the estimates using the maximum likelihood method (Jöreskog and Sörbom, 1996), the linearity suppositions must be met and all the observed variables included in the model must follow a normal distribution pattern. The residual scatter plots made reflected the fact that there is linearity between the estimated variables. After eliminating some atypical scores, the sample data did comply with the normality criterion.

Once the atypical scores had been eliminated, the CFA was conducted using a sample of *n* = 680. The aim was to confirm whether the QTLG follows the same factorial model as the *achievement goal questionnaire* (3 × 2 AGQ) of Elliot et al. (2011).

Table 1 shows the 12 models used in the procedure applied by Elliot et al. (2011) to compare the fit of their hypothetical model (3 × 2 AGQ).

**TABLE 1:** Models subjected to confirmatory analysis.

	**Model**	**No. of factors**	**Factors**		
1	*3* × *2 our base hypothetical model*	3 second order	F1 task	F1 approach-task	F2 avoidance-task
		6 first order	F2 self	F3 approach-self	F4 avoidance-self
			F3 other	F5 approach-other	F6 avoidance- other
2	*3* × *2*	6 factors	F1 approach-task	F2 avoidance-task	
			F3 approach-self	F4 avoidance-self	
			F5 approach-other	F6 avoidance-other	
3	*ApT/AvT*	5 factors	F1 approach-avoidance-task		
			F2 approach-self	F3 avoidance-self	
			F4 approach-other	F5 avoidance-other	
4	*ApO/AvO*	5 factors	F1 approach-task	F2 avoidance-task	
			F3 approach-self	F4 avoidance-self	
			F5 approach-avoidance-other		
5	*ApE/AvE*	5 factors	F1 approach-task	F2 avoidance-task	
			F3 approach-avoidance-self		
			F4 approach-other	F5 avoidance-other	
6	*Definition*	3 factors	F1 task- approach-avoidance		
			F2 self approach-avoidance		
			F3 other- approach-avoidance		
7	*2* × *2*	4 factors	F1 approach-other	F2 avoidance-other	
			F3 approach-task-self	F4 avoidance -task-self	
8	*Trichotomous*	3 factors	F1 approach-other	F2 avoidance-other	F3 task-self
9	*Dichotomous*	2 factors	F1 other	F2 task-self	
10	*Avoidance*	4 factors	F1 approach-task	F2 approach-self	
			F3 approach-other	F4 avoidance-task-self-other	
11	*Approach*	4 factors	F1 avoidance-task	F2 avoidance-self	
			F3 avoidance-other	Approach-task-self-other	
12	*Valence*	2 factors	F1 approach-task-self-other		
			F2 avoidance-task-self-other		

Finally, we compared twelve models. We analyzed the 18 items of the QTLG. Table 2 shows the statistics for the goodness of fit of the CFA considering the models used in the procedure applied by Elliot et al. (2011) to compare the fit of their hypothetical model (3 × 2 AGQ).

**TABLE 2:** Goodness-of-fit indexes of the proposed models.

**Models**	**χ^2^**	**CMIN/*df***	**CFI**	**TLI**	**RMSEA**	**SRMR**	**Δχ^2^**	**AIC**	**BCC**
M1: Model 3 × 2	375,57	3.078	0.955	0.950	0.056	0.047		473.57	476.46
M2: Model 3 × 2	625.34	5.391	0.910	0.881	0.081	0.086	249.77	735.34	738.59
M3: Model ApT/AvT	752.63	6.021	0.889	0.864	0.087	0.095	350.06	844.63	847.35
M4: Model ApO/AvO	766.17	6.129	0.887	0.862	0.088	0.088	390,60	858.17	860.89
M5: Model ApY/AvY	817.40	6.539	0.878	0.850	0.091	0.090	441.83	909.40	912.12
M6: Model definition	898.67	6.808	0.865	0.843	0.094	0.103	523.10	976.67	978.98
M7: Model 2 × 2	1360.32	10.545	0.783	0.742	0.120	0.082	984.75	1444.32	1446.80
M8: Trichotomous model	1363.40	10.652	0.782	0.739	0.121	0.082	987.83	1449.40	1451.94
M9: Dichotomous model	1487.43	11.100	0.761	0.727	0.124	0.087	1111.86	1561.62	1764.81
M10: Avoidance model	1980.27	15.351	0.673	0.613	0.147	0.109	1604.47	2064.27	2066.75
M11: Approach model	2161.25	16.754	0.641	0.575	0.154	0.116	1785.68	2245.25	2247.73
M12; Valence model	2592.17	19.345	0.566	0.505	0.166	0.123	2216.60	2666.17	2668.36

**FIGURE 1:**
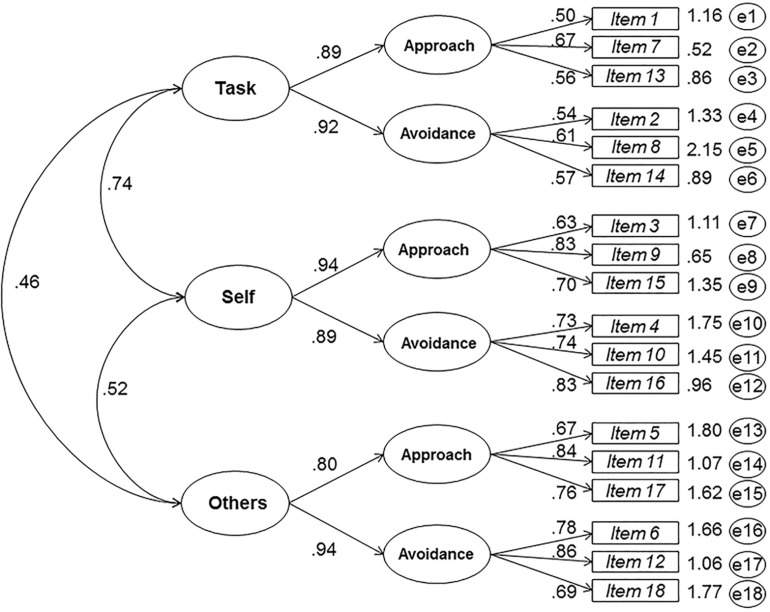
The 3 × 2 model of 6 first order factors and 3 related second order factors with the questionnaire on team work learning goals (QTLG).

All the models present a significant value of χ2 (*p* < 0.001) and although large sample sizes of χ2 tend to be statistically significant, from a practical perspective, it is more convenient to take into account the magnitude of the value of χ2 or χ2 divided by the degrees of freedom (CMIN/DF) than the level of statistical significance. Large values would correspond to a poor fit and small values to a good fit. The models “M2-M12” have all been discarded. The fitness indicators CFI and TLI are expected to be greater than or equal to 0.95, a value which is not achieved in these models. The expected values of the RMSEA and SRMR indicators are not reached either, as these are expected to be lower than 0.06 and 0.08, respectively. The values that best fit what is expected can only be found in the model M1, which is represented in Figure 1; in addition, this model has lower AIC and BCC values than all the remaining alternative models.

The results of the model indicate that the second order factors Task, Self and Other are correlated (Other and Self β = 0.52; Self and Task β = 0.74; Other and Task β = 0.46). On the other hand, the indicators of the latent factors show factorial loads that range between λ = 0.50 and ανδλ = 0.86. This implies that the factors are well defined and, thus, the way in which they have been evaluated is adequate. In addition, values higher than 0.50 of AVE, 0.80 of Composite Reliability and McDonald’s Omega coefficient (Table 3) corresponding to the model M1 of 6 first-order factors and three related second-order factors, show evidence of the questionnaire’s reliability.

**TABLE 3:** Values of AVE, CR, and Ω of the QTLG scores.

	**Total QTLG**	**approach Task**	**Avoid task**	**Approach self**	**Avoid self**	**Approach other**	**Avoid other**
Average variance extracted	0.493	0.328	0.329	0.525	0.590	0.577	0.608
Composite reliability	0.945	0.589	0.595	0.766	0.811	0.803	0.822
McDonald’s omega	0.889	0.613	0.576	0.765	0.816	0.805	0.816
Standardized Cronbach’s alpha	0.890	0.604	0.574	0.762	0.814	0.805	0.811

As can be seen in Table 4, using a total of 1,000 samples, we have obtained some averages for the factorial saturation values very close to those found in the confirmatory analysis. On the other hand, it can also be seen that the factorial saturation values are between the lower and upper limits of the CI at 95%, thus all being significant.

**TABLE 4:** Bootstrap method, 1,000 samples with a CI at 95%.

	**Parameter**	**Estimate**	**Average 1000 samples**	**Lower**	**Upper**	***p***
Approach task	Item 1	0.504	0.473	0.391	0.561	0.002
	Item 7	0.670	0.670	0.581	0.746	0.003
	Item 13	0.562	0.561	0.477	0.650	0.002
Avoidance task	Item 2	0.544	0.546	0.456	0.627	0.003
	Item 8	0.607	0.604	0.530	0.684	0.001
	Item 14	0.567	0.567	0.475	0.640	0.003
Approach self	Item 3	0.628	0.630	0.545	0.697	0.004
	Item 9	0.826	0.827	0.777	0.865	0.004
	Item 15	0.704	0.702	0.635	0.765	0.002
Avoidance self	Item 4	0.739	0.739	0.671	0.800	0.003
	Item 10	0.830	0.830	0.775	0.874	0.002
	Item 16	0.728	0.727	0.665	0.778	0.002
Approach other	Item 5	0.665	0.664	0.612	0.713	0.002
	Item 11	0.841	0.841	0.800	0.880	0.002
	Item 17	0.756	0.755	0.700	0.803	0.002
Avoidance other	Item 6	0.779	0.778	0.730	0.823	0.002
	Item 12	0.858	0.859	0.811	0.892	0.004
	Item 18	0.688	0.686	0.630	0.740	0.002

Next, we carried out a multi-group analysis to determine whether the model of two related factors is invariant by gender (577 women and 123 men). The comparison shows no differences *p* < 0.05 in the value of χ2 between the different models and the values found in the ΔCFI in the model without restrictions had differences below 0.01 of the CFI indices between the four models, indicating that the factorial loads of the questionnaire are equivalent for both men and women (Table 5).

**TABLE 5:** Multi-group analysis of invariance by gender.

**Models**	**χ2**	**gl**	**χ2/gl**	**Δχ2**	***p***	**Δgl**	**CFI**	**TLI**	**SRMR**	**RMSEA**
Model 1	552.328	244	2.264	−		−	0.946	0.932	0.046	0.044
Model 2	560.000	256	2.187	7,672	0.810	12	0.947	0.936	0.047	0.042
Model 3	561.842	259	2.169	9.514	0.849	15	0.947	0.937	0.049	0.042
Model 4	518.036	265	2.181	25.709	0.218	21	0.945	0.937	0.049	0.042

*Model 1, without restrictions; Model 2, measurement weights; Model 3, structural covariances; Model 4, measurement residuals.*

### Nomological Validity of the QTLG

In order to check whether there is a correspondence between the theoretical configuration of the data obtained and the theoretical predictions concerning the said configuration, we have related the factors of the QTLG with those of the QALT (Mendo et al., 2017) and we have carried out a regression analysis considering the factors of the QTLG as predictive variables and the Confidence factor of the LTPQ (León et al., 2017) as the dependent variable. Thus, the relationships that a construct can maintain with others that make up, either totally or partially, some theory or theories can be empirically demonstrated (Wilson et al., 1989).

Table 6 shows the direct/low correlations between most of the factors of the QTLG with the QALT, except for academic attitudes with task avoidance and social attitudes with self and other avoidance.

**TABLE 6:** Pearson correlations between QTLG, QALT factors, and QPLT factors.

		**Task**		**Self**		**Other**	
		**Approach**	**Avoidance**	**Approach**	**Avoidance**	**Approach**	**Avoidance**
QALT	Academic attitudes	0.126^∗∗^	0.038	0.221^∗∗^	0.125^∗∗^	0.135^∗∗^	0.115^∗∗^
	Social attitudes	0.277^∗∗^	0.125^∗∗^	0.218^∗∗^	0.062	−0.006	0.071

*^∗∗^The correlation is significant at the 0.01 level.*

The regression model predicts 20.0% of the variance of the Confidence factor; therefore, the model is significant (*F* = 18.435; *p* = 0.000). The overall relationship between the model and the dependent variable is significant at *p* < 0.001. As can be seen in Table 7, the factors Approach-Task (β = 0.169; *t* = 3.127; *p* = 0.002), and Approach-Self (β = 0.160; *t* = 2.971; *p* = 0.003), present a highly predictive capacity over the beliefs concerning the effectiveness of the team. On the other hand, the factors Avoidance-Task, Avoidance-Self, Approach-Other, or Avoidance-Other do not have this predictive capacity.

**TABLE 7:** Coefficients of the regression model for predicting the confidence factor of the questionnaire on the power of learning in teams (QPLT) from the different factors of the QTLG.

	**Not standardized coefficients**		**Typified coefficients**		
	***B***	**Typical error**	**β**	***t***	***p***
Constant	21.328	1.875		11.378	<0.001
Approach task	0.368	0.118	0.169	3.127	0.002
Approach self	0.229	0.077	0.160	2.971	0.003

*Successive step method.*

## Discussion

Following the idea proposed by Elliot et al. (2011) of applying the 3 × 2 model to other, different evaluative environments, we decided to analyze the academic goals when working in teams. Thus, we adapted the *achievement goal questionnaire* (3 × 2 AGQ) and created the QTLG, which has some highly acceptable psychometric characteristics.

The confirmatory analysis carried out has demonstrated the existence of six solid, well-defined factors corresponding to the six academic achievement goals. The weights or loads of the items that define these six factors have values above 0.50. For Costello and Osborne (2005), when a factor is defined by 3–5 items with weights over 0.50, it is a solid factor with practical relevance. On the other hand, the analysis by means of structural equations and the application of the bootstrap method has allowed us to verify that the values of the factorial loads are between the upper and lower limits of the CI at 95%, all of them being significant. Thus, the latent variables in the six factors are well defined and the way in which they have been evaluated is adequate, confirming the good psychometric characteristics of the scale.

Our results confirm the 3 × 2 model with six first-order factors and three second-order factors in the context of teamwork, since this model displays a better fit to the data than the rest of the models subjected to the confirmatory analysis, including the 3 × 2 model with six related factors (2 × 2, Dichotomous, Trichotomous, Approach, Avoidance, Definition, Valence, ApT/AvT, ApE/AvE, and ApO/AvO). We feel we should stress the importance of finding the same tendency in the fit of the alternative models as in the work of Elliot et al. (2011). The models with the worst fit are Avoidance, Definition, and Valence. The ApT/AvT, ApE/AvE and ApO/AvO models present the best fit after the 3 × 2 models. These same tendencies have been found recently by Méndez-Giménez et al. (2017) on examining the structural validity of the Spanish version of the *achievement goal questionnaire* (3 × 2 AGQ) of Elliot et al. (2011) with a sample of 2,630 non-university Spanish students studying in secondary schools and sixth-form colleges. In short, the 3 × 2 model maintains its structural validity regarding the goals adopted by the students when faced with working in teams, an evaluative environment different from that which the students face in their exams of a specific subject.

Harackiewicz et al. (2000) argue that the goals the students have depend on personal variables, as they can be considered stable constructs of a dispositional type. Gender has been studied as a factor influencing this type of goal (Shibley and Durik, 2005). Research into this aspect has shown that the intensity of the different types of goals depends on gender. Males tend toward work avoidance, while females tend toward learning (Lightbody et al., 1996; Chaplain, 2000). To ensure that, in future research using the QTLG, the differences between males and females are due to real differences in the evaluated construct and not to different psychometric responses to the questionnaire’s items (Cheung and Rensvold, 2002; Pedraza and Mungas, 2008), we carried out a multi-group analysis to determine whether the 3 × 2 model of related factors was invariant by gender. The results obtained confirm the equality between males and females in their perception of the evaluated construct. The data support the equivalence of the factorial structure of the QTLG with respect to gender.

In addition, the analyses carried out on the QTLG, the QALT and the LTPQ confirm their nomological validity (Cronbach and Meehl, 1955). The attitudes toward teamwork and team potency are two relevant, motivational variables which determine the effectiveness of the teamwork (León et al., 2017; Mendo et al., 2017). On the one hand, the positive attitude toward teamwork is essential, it being one of the mechanisms involved in the team’s positive results, and it can only be developed if the individualistic, competitive orientation is left to one side (Castelló, 1998). To do so involves abandoning the belief that success depends solely on one’s own efforts and requires trust in the ability of the teammates.

The correlations between the QTLG and QALT factors confirm the relationship between goals toward teamwork and attitudes, suggesting that those students participating in the study with academic attitudes, i.e., those that value the academic consequences derived from teamwork positively (academic attitudes factor) maintain, with a greater intensity, their teamwork goals in Self, Other and Approach-Task. Nevertheless, when students assess their interactions with their teammates during the work positively (Social and Affective Attitudes Factor), they display less intensity regarding the goals related to Avoidance-Self and Other.

Although, in general, attitudes toward teamwork can determine an individualistic or collaborative orientation among students (Mendo et al., 2017), those with social and affective attitudes, i.e., those who feel more useful and valued by their peers, feel at ease and trust them, would not aim to compete, or would avoid competing, with their peers when working in a team.

On the other hand, team potency is a concept originally defined by Guzzo et al. (1993). It refers to the existing collective beliefs in the group with respect to whether it can be effective and is an essential construct, related with the group’s motivation, which can improve the attitude of the team members in order to successfully carry out a task, as well as the capacity to solve problems that may arise during teamwork. Regression analysis, considering the factors of the QTLG as predictive variables and the Confidence factor in the LTPQ as a dependent variable, indicates that students participating in the study who face teamwork scenarios with Approach-Task and Approach-Self goals maintain positive expectations concerning the effectiveness of their own team (Confidence factor). As in other research, the Approach-Task and Approach-Self goals turn out to be more adaptable and positively predict motivation (Church et al., 2001; Elliot et al., 2011; Méndez-Giménez et al., 2017) and efficiency in the task (Elliot et al., 2005).

Although the QTLG presents preliminary evidence of validity and reliability, it is not exempt from limitations, such as the difficulty of extrapolating the results to other university population groups, which compromise the questionnaire’s external validity (concerning population and ecology), or of establishing sufficient evidence of the QTLG’s convergent and discriminatory validity. As future lines of research, in addition to finding an answer to the limitations set out here, it would be of interest to explore the validity of the QTLG among the non-university population. In addition, in the line of previous studies (Michou et al., 2014; Sommet and Elliot, 2017), we believe it would be interesting to study the reasons underlying the pursuit of achievement goals (e.g., personal challenge; pressure; gaining respect from others; the desire to experience pride, instructor demand).

## Conclusion

In conclusion, we have a useful instrument for the study of motivation in the context of learning through teamwork. The QTLG has practical applications, allowing us to explore, in depth, students’ academic goals. As well as training in methodologies that favor cooperation, management planning, and teamwork evaluation (León et al., 2015), prior knowledge of students’ goals when forming learning teams can be useful for the instructor at the time of creating or putting together more successful and effective teams (Pujolàs, 2008). However, teamwork is not always a satisfactory experience for university students (Payne and Monk-Turner, 2006; Burdett, 2007), thus, designing tasks and/or dynamics adjusted to the student’s profile can help to prevent or address possible difficulties or conflicts of interest within the teams. In addition, the QTLG allows students to evaluate their own learning goals, reflect on how certain goals impact teamwork and develop academic goals that serve the common good.

## Data Availability Statement

All datasets generated for this study are included in the article/Supplementary Material.

## Ethics Statement

This study received approval from the Ethics Committee of the University of Extremadura. The questionnaires were filled in anonymously, guaranteeing the confidentiality of the data obtained, and their exclusive use for research. Insofar as ethical rules are concerned, we followed the ethical directives of the American Psychological Association (APA, 2009) concerning the participants’ informed consent.

## Author Contributions

BL-d-B and SM-L: analysis and interpretation of the data, and drafting the work. BL-d-B, SM-L, MP-d-R, and IR-G: conception and design of the work. All authors listed have made a substantial, direct and intellectual contribution to the work, and approved it for publication.

## Conflict of Interest

The authors declare that the research was conducted in the absence of any commercial or financial relationships that could be construed as a potential conflict of interest.

## References

[B1] Alford L. K. Fowler R. Sheffield S. (2014). “Evolution of student attitudes toward teamwork in a project-based, team-based first-year introductory engineering course,” in *Paper Presented in 2014 at the ASEE Annual Conference*, Indianapolis, IN.

[B2] APA (2009). *Publication Manual of the American Psychological Association*, 6th Edn., Worcester, MA: American Psychological Association.

[B3] Atkinson. (1964). *An Introduction to Motivation.* Princeton, NJ: Van Nostrand.

[B4] Beigi M. Shirmohammadi M. (2012). Attitudes toward teamwork: are iranian university students ready for the workplace? *Team Perform. Manag.* 18 295–311. 10.1108/13527591211251087

[B5] Blignaut R. J. Venter I. M. (1998). Teamwork: can it equip university science students with more than rigid subject knowledge? *Comp. Educ.* 31 265–279. 10.1016/s0360-1315(98)00031-1

[B6] Brophy J. (2005). Goal theorists should move on from performance goals. *Educ. Psychol.* 40 167–176. 10.1207/s15326985ep4003_3

[B7] Burdett J. (2007). Degrees of separation — balancing intervention and independence in group work assignments. *Aust. Educ. Res.* 34 55–71. 10.1007/bf03216850

[B8] Castelló T. (1998). “Procesos de cooperación en el aula [cooperation processes in the classroom],” in *Cooperar en la Escuela. La Responsabilidad de Educar Para la Democracia [Cooperate in School. The Responsibility of Educating for Democracy]*, ed. Mir C. (Barcelona: Graó).

[B9] Chaplain R. P. (2000). Beyond exam results? differences in the social and psychological perceptions of young males and females at school. *Educ. Stud.* 26 177–190. 10.1080/713664271

[B10] Cheung G. W. Rensvold R. B. (2002). Evaluating goodness-of-fit indexes for testing measurement invariance. *Struct. Equa. Model.* 9 233–255. 10.1097/NNR.0b013e3182544750 22551991 PMC3361901

[B11] Church M. A. Elliot A. J. Gable S. L. (2001). Perceptions of classroom environment, achievement goals, and achievement outcomes. *J. Educ. Psychol.* 93 43–54. 10.1037//0022-0663.93.1.43

[B12] Collins C. G. Parker S. K. (2010). Team capability beliefs over time: distinguishing between team potency, team outcome efficacy, and team process efficacy. *J. Occup. Organ. Psychol.* 83 1003–1023. 10.1348/096317909x484271

[B13] Costello A. B. Osborne J. W. (2005). Best practices in exploratory factor analysis: four recommendations for getting the most from your analysis. *Pract. Assess. Res. Eval.* 10 1–9.

[B14] Cronbach L. J. Meehl P. E. (1955). Construct validity in psychological tests. *Psychol. Bull.* 52 281–300. 13245896 10.1037/h0040957

[B15] Dweck C. S. (1986). Motivational processes affecting learning. *Am. Psychol.* 41 1040–1048. 10.1037//0003-066x.41.10.1040

[B16] Elliot A. J. (1999). Approach and avoidance motivation and achievement goals. *Educ. Psychol.* 34 169–189. 10.1207/s15326985ep3403_3

[B17] Elliot A. J. (2005). “A conceptual history of the achievement goal construct,” in *Handbook of Competence and Motivation*, eds Elliot A. Dweck C. (New York, NY: J. P Guilford).

[B18] Elliot A. J. Harackiewicz J. M. (1996). Approach and avoidance achievement goals and intrinsic motivation: a mediational analysis. *J. Personal. Soc. Psychol.* 70 461–475. 10.1037//0022-3514.70.3.461 8014838

[B19] Elliot A. J. McGregor H. A. (2001). A 2 × 2 achievement goal framework. *J. Personal. Soc. Psychol.* 80 501–519. 10.1037/-0022-3514.80.3.501 11300582

[B20] Elliot A. J. Murayama K. Pekrun R. (2011). A 3 × 2 achievement goal model. *J. Educ. Psychol.* 103 632–648. 10.1037/-a0023952

[B21] Elliot A. J. Shell M. M. Henry K. B. Maier M. A. (2005). Achievement goals, performance contingencies, and performance attainment: an experimental test. *J. Educ. Psychol.* 97 630–640. 10.1037/0022-0663.97.4.630

[B22] Gaudet A. D. Ramer L. M. Nakonechny J. Cragg J. J. Ramer M. S. (2010). Small-group learning in an upper-level university biology class enhances academic performance and student attitudes toward group work. *PLoS One* 5:e15821. 10.1371/journal.pone.0015821 21209910 PMC3012112

[B23] González M. Raposo M. (2008). Necesidades formativas del profesorado universitario en el contexto de la convergencia europea. *Rev. Invest. Educ.* 26 285–306.

[B24] Gottschall H. García-Bayonas M. (2008). Student attitudes towards group work among undergraduates in business administration, education and mathematics. *Educ. Res. Q.* 32 3–28.

[B25] Gully S. M. Incalcaterra K. A. Joshi A. Beaubien J. M. (2002). A meta-analysis of team efficacy, potency, and performance: interdependence and level of analysis as moderators of observed relationships. *J. Appl. Psychol.* 87 819–832. 10.1037//0021-9010.87.5.819 12395807

[B26] Guzzo R. A. Yost P. R. Campbell R. J. Shea G. P. (1993). Potency in groups: articulating a construct. *Br. J. Soc. Psychol.* 32 87–106. 10.1111/j.2044-8309.1993.tb00987.x 8467372

[B27] Harackiewicz J. M. Barron K. E. Tauer J. M. Carter S. M. Elliot A. J. (2000). Short - term and long - term consequences of achievement goals: predicting interest and performance over time. *J. Educ. Psychol.* 92 316–330. 10.1037//0022-0663.92.2.316

[B28] Henson R. K. Roberts J. K. (2006). Use of exploratory factor analysis in published research: common errors and some comment on improved practice. *Educ. Psychol. Measure.* 66 393–416. 10.1177/00131-64405282485

[B29] Hijzen D. Boekaerts M. Vedder P. (2006). The relationship between the quality of cooperative learning, students’ goal preferences, and perceptions of contextual factors in the classroom. *Scand. J. Psychol.* 47 9–21. 10.1111/j.1467-9450.2006.00488.x 16433658

[B30] Huang C. (2012). Discriminant and criterion-related validity of achievement goals in predicting academic achievement: a meta-analysis. *J. Educ. Psychol.* 104 48–73. 10.1037/a0026223

[B31] Johnson D. W. Johnson R. T. Holubec E. J. (1999). *El Aprendizaje Cooperativo en el Aula.* Paidós: Barcelona.

[B32] Jöreskog K. G. Sörbom D. (1996). *Lisrel 8: User’s Reference Guide.* Chicago: SSI.

[B33] Kouros C. Abrami P. C. (2006). “How do students really feel about working in small groups? the role of student attitudes and behaviors in cooperative classroom settings,” in *Proceedings of the Annual Meeting of the American Educational Research Association*, San Francisco, CA.

[B34] León B. Felipe E. Mendo S. Iglesias D. (2015). Habilidades sociales en equipos de aprendizaje cooperativo en el contexto universitario. *Behav. Psychol.* 23 191–214.

[B35] León B. Mendo S. Felipe E. Fajardo F. Iglesias D. (2018). Measuring responsibility and cooperation in learning teams in the university setting: validation of a questionnaire. *Front. Psychol.* 9:326. 10.3389/fpsyg.2018.00326 29593622 PMC5859103

[B36] León B. Mendo S. Felipe E. Polo M. I. Fajardo F. (2017). Team potency and cooperative learning in the university setting. *Rev. Psicodidáctica* 22 9–15. 10.1387/Rev-Psicodidact.14213 29593622

[B37] Lightbody P. Siann G. Stocks R. Walsh D. (1996). Motivation and attribution at secondary school: the role of gender. *Educ. Stud.* 22 13–25. 10.1080/0305569960220102

[B38] Locke E. A. Latham G. P. (1990). *A Theory of Goal Setting & Task Performance.* Englewood Cliffs, NJ: Prentice-Hall, Inc., 413.

[B39] Lower L. M. Turner B. A. (2016). Examination of the 3x2 achievement goal model in collegiate recreation: comparison across sport programs. *J. Amateur Sport* 2 75–102.

[B40] Manzer J. Bialik D. (1997). Team and group learning strategies for business and economics classes. *Bus. Educ. Forum* 151 32–35.

[B41] Mega C. Ronconi L. De Beni R. (2014). What makes a good student? How emotions, self-regulated learning, and motivation contribute to academic achievement. *J. Educ. Psychol.* 106 121–131. 10.1037/a0033546

[B42] Mena B. Barrasa Á Gil F. (2012). Análisis de la influencia de la interdependencia y la potencia grupal en la eficacia de los equipos de trabajo en contextos sanitarios. *Rev. Psicol. Soc.* 27 111–122. 10.1174/021347412798844006

[B43] Méndez-Giménez A. Cecchini-Estrada J. A. Fernández-Río J. Méndez-Alonso D. Prieto-Saborit J. A. (2017). 3 × 2 Achievement goals, self-determined motivation and life satisfaction in secondary education. *Rev. Psicodidv. Psicodidáctica* 22 150–157.

[B44] Mendo S. León B. Felipe E. Pol M. I. Palacios V. (2016). Assessment of social skills of students of social education. *Rev. Psicodidáctica* 21 139–156. 10.1387/Rev-Psicodidact.14031

[B45] Mendo S. Polo M. I. Iglesias D. Felipe E. León B. (2017). Construction and validation of a measurement instrument for attitudes towards teamwork. *Front. Psychol.* 8:1–10. 10.3389/fpsyg.2017.01009 28676775 PMC5477521

[B46] Mercier E. M. (2017). The influence of achievement goals on collaborative interactions and knowledge convergence. *Learn. Inst.* 50 31–43. 10.1016/j.learninstruc.2016.11.006

[B47] Michou A. Vansteenkiste M. Mouratidis A. Lens W. (2014). Enriching the hierarchical model of achievement motivation: autonomous and controlling reasons underlying achievement goals. *Br. J. Educ. Psychol.* 84 650–666. 10.1111/bjep.12055 25251866

[B48] Midgley C. Kaplan A. Middleton M. (2001). Performance-approach goals: good for what, for whom, under what circumstances, and at what cost? *J. Educ. Psychol.* 93 77–86. 10.1037/0022-0663.93.1.77

[B49] Murayama K. Elliot A. J. Yamagata S. (2011). Separation of performance-approach and performance-avoidance achievement goals: a broader analysis. *J. Educ. Psychol.* 103 238–256. 10.1037/a0021948

[B50] Nausheen M. Alvi E. Munir S. Anwar R. (2013). Attitudes of postgraduate students towards cooperative learning. *J. Educ. Res.* 16 107–115. 16635120

[B51] Nicholls J. G. Miller A. T. (1984). Reasoning about the ability of self and others: a developmental study. *Child Dev.* 55 1990–1999. 10.1111/j.1467-8624.1984.tb03897.x 10384736

[B52] Palacios A. (2004). El crédito europeo como motor de cambio de la configuración del espacio europeo de la educación superior. *Rev. Int. Formación Del Prof.* 18 197–205.

[B53] Payne B. K. Monk-Turner E. (2006). Students’ perceptions of group projects: the role of race, age, and slacking. *College Stud. J.* 40 132–139.

[B54] Pedraza O. Mungas D. (2008). Measurement in cross-cultural neuropsychology. *Neuropsychol. Rev.* 18 184–193. 10.1007/s11065-008-9067-9 18814034 PMC2925437

[B55] Pfaff E. Huddleston P. (2003). Does it matter if i hate teamwork? what impacts student attitudes toward teamwork. *J. Market. Educ.* 25 37–45. 10.1177/0273475302250571

[B56] Prichard J. S. Stratford R. J. Bizo L. A. (2006). Team-skills training enhances collaborative learning. *Learn. Instruct.* 16 256–265. 10.1016/j.learninstruc.2006.03.005

[B57] Pujolàs P. (2008). *9 Ideas Clave. El aprendizaje Cooperativo.* Barcelona: Editorial Graó.

[B58] Rico R. Alcover C. M. Tabernero C. (2010). Efectividad de los equipos de trabajo: una revisión de la última década de investigación (1999-2009). *Rev. Psicol. Trabajo Organ.* 26 47–71. 10.5093/tr2010v26n1a4

[B59] Rodríguez F. J. Ridao S. (2014). El trabajo en equipo como recurso para fomentar las habilidades sociales en estudiantes universitarios. *Educ. Futuro* 31 273–288.

[B60] Shibley J. Durik A. M. (2005). “Gender, competence, and motivation,” in *Handbook of Competence and Motivation*, eds Dweck C. Y. Elliot A. (New York, NY: Guilford), 375–391.

[B61] Shim S. S. Cho Y. Wang C. (2013). Classroom goal structures, social achievement goals, and adjustment in middle school. *Learn. Instruct.* 23 69–77. 10.1016/j.learninstruc.2012.05.008

[B62] Sommet N. Elliot A. J. (2017). Achievement goals, reasons for goal pursuit, and achievement goal complexes as predictors of beneficial outcomes: is the influence of goals reducible to reasons? *J. Educ. Psychol.* 109 1141–1162. 10.1037/edu0000199

[B63] Wilson P. H. Spence S. H. Kavanagh D. J. (1989). *Cognitive-Behavioral Interviewing for Adult Disorders: A Practical Handbook.* London: Routledge.

