# Supplementary material for: University Student’s Academic Goals When Working in Teams: Questionnaire on Academic Goals in Teamwork, 3 × 2 Model
Source: Front Psychol. 2019 Oct 24;10:2434. doi: 10.3389/fpsyg.2019.02434 (PMC6821790; doi:10.3389/fpsyg.2019.02434)
Supplement: Supplementary file 1 [file Table_1.DOCX]

**Supplementary material**

**Questionnaire on Teamwork Goals**

The following phrases represent the desired goals when working in a team.

Please answer honestly, circling the number that is closest to what you believe.

When I do a task in a group, my goals are to…

1 (not at all true for me) to 7 (very true for me).

| 1 have many tasks well done in all the subjects | 1 | 2 | 3 | 4 | 5 | 6 | 7 |
| --- | --- | --- | --- | --- | --- | --- | --- |
| 2 avoid having tasks badly done in all the subjects | 1 | 2 | 3 | 4 | 5 | 6 | 7 |
| 3 perform better than I did in the past | 1 | 2 | 3 | 4 | 5 | 6 | 7 |
| 4 avoid doing worse than I normally do | 1 | 2 | 3 | 4 | 5 | 6 | 7 |
| 5 do better than the other students | 1 | 2 | 3 | 4 | 5 | 6 | 7 |
| 6 avoid doing worse than the other students | 1 | 2 | 3 | 4 | 5 | 6 | 7 |
| 7 know how to do the tasks well | 1 | 2 | 3 | 4 | 5 | 6 | 7 |
| 8 avoid not knowing how to do the tasks | 1 | 2 | 3 | 4 | 5 | 6 | 7 |
| 9 do the tasks well in comparison with how I did them in the past | 1 | 2 | 3 | 4 | 5 | 6 | 7 |
| 10 avoid doing them worse than my normal level of performance | 1 | 2 | 3 | 4 | 5 | 6 | 7 |
| 11 do the tasks well in comparison with the other students | 1 | 2 | 3 | 4 | 5 | 6 | 7 |
| 12 avoid doing the tasks worse than the other students | 1 | 2 | 3 | 4 | 5 | 6 | 7 |
| 13 resolve the doubts I have in order to do the tasks well | 1 | 2 | 3 | 4 | 5 | 6 | 7 |
| 14 resolve the doubts I have in order to avoid doing the tasks badly | 1 | 2 | 3 | 4 | 5 | 6 | 7 |
| 15 do the tasks better than I usually do | 1 | 2 | 3 | 4 | 5 | 6 | 7 |
| 16 avoid doing the tasks worse than I have done before | 1 | 2 | 3 | 4 | 5 | 6 | 7 |
| 17 do the tasks better than the other teams | 1 | 2 | 3 | 4 | 5 | 6 | 7 |
| 18 avoid a deficient performance in comparison to my companions | 1 | 2 | 3 | 4 | 5 | 6 | 7 |
